# Supplementary material for: Role of Infodemics on Social Media in the Development of People’s Readiness to Follow COVID-19 Preventive Measures
Source: Int J Environ Res Public Health. 2022 Jan 25;19(3):1347. doi: 10.3390/ijerph19031347 (PMC8834964; doi:10.3390/ijerph19031347)
Supplement: Supplementary file 1 [file ijerph-19-01347-s001.zip › ijerph-1469032-supplementary.pdf]

Table S1. Constructs and items.

Constructs	Code	Items	Source
Attitude	ATT1	Following COVID-19 preventive measures is a good idea	Dutta et al. [43]; Hwang et al. [44]
	ATT2	Following COVID-19 preventive measures is pleasant.	
	ATT3	Following COVID-19 preventive measures is beneficial	
Trust in the Government	TRGT1	I believe the government	Enria et al. [33]; Kapoor et al. [34]
	TRGT2	I consider the government is trustworthy regarding COVID-19 preventive measures	
	TRGT3	I sense most things the government does regarding COVID-19 prevention are correct	
Trust in Social media	TRSM1	I believe the news circulated on social media regarding the preventive measurements of COVID-19	Enria et al. [33]; Kapoor et al. [34]
	TRSM2	I feel most of the information circulated on social media regarding COVID-19 prevention are correct	
	TRSM3	In total, social media is trustworthy regarding the news of COVID-19	
Perceived benefit	PBT1	COVID-19 preventive measures are convenient to follow	Gong et al. [47]; Hong et al. [49]
	PBT2	Following COVID-19 preventive measures can improve my health	
	PBT3	Following COVID-19 preventive measures can improve my productivity in terms of improving my health	
Personal innovativeness	PIIT1	If I heard about a new regulation, COVID-19 preventive measures, I look for ways to follow	Sun et al. [11]; Alkawsi et al. [12]
	PIIT2	I like to experiment to follow COVID-19 preventive measures	
	PIIT3	Generally, I would not be hesitant to follow COVID-19 preventive measures	
Peer referent	PRT1	People I know think I should follow COVID-19 preventive measures.	Potarca [9]; Sakallaris et al. [52]
	PRT2	Social media believe that the public should follow COVID-19 preventive measures.	
	PRT3	Insofar as I know, everyone around me is following COVID-19 preventive measures.	
Health infodemic	HID1	I am influenced by interpretations, videos, and comments made on social media about COVID-19 preventive measures.	Kim et al. [18]; Cinelli and Galeazzi [19]
	HID2	I believe whatsoever is accessible on social media is true about COVID-19 preventive measures.	
	HID3	Social media misguides people about health entities regarding COVID-19 preventive measures.	
	HID4	I believe most of the content presented on social media is false about COVID-19 preventive measures.	
	HID5	Social media shape my personal opinions about COVID-19 preventive measures.	
Readiness toward COVID-19 preventive measures	INT1	I intend to follow COVID-19 preventive measures	Dutta et al. [43]; Hwang et al. [44]
	INT2	My readiness toward COVID-19 preventive measures is positive.	
	INT3	The probability that I follow COVID-19 preventive measures daily is very high	
	INT4	Whatsoever the situations, I do not intend to toward COVID-19 preventive measures	
